# Morphological and genetic characterization of an emerging Azorean horse breed: the Terceira Pony

**DOI:** 10.3389/fgene.2015.00062

**Published:** 2015-02-27

**Authors:** Maria S. Lopes, Duarte Mendonça, Horst Rojer, Verónica Cabral, Sílvia X. Bettencourt, Artur da Câmara Machado

**Affiliations:** Biotechnology Centre of Azores, Department of Agriculture, University of Azores, Angra do HeroísmoPortugal

**Keywords:** Azores, equine, morphotype, SSR, genetic diversity, management, conservation

## Abstract

The Terceira Pony is a horse indigenous to Terceira Island in the Azores. These horses were very important during the colonization of the island. Due to their very balanced proportions and correct gaits, and with an average withers height of 1.28 m, the Terceira Pony is often confused with a miniature pure-bred Lusitano. This population was officially recognized as the fourth Portuguese equine breed by the national authorities in January, 2014. The aim of this study was to analyze the morphology and the genetic diversity by means of microsatellite markers of this emerging horse breed. The biometric data consisted of 28 body measurements and nine angles from 30 animals (11 sires, 19 dams). The Terceira Pony is now a recognized horse breed and is gaining in popularity amongst breeders and the younger riding classes. The information obtained from this study will be very useful for conservation and management purposes, including maximizing the breed’s genetic diversity, and solidifying the desirable phenotypic traits.

## INTRODUCTION

Horses have played an important role in the development of Portugal throughout the centuries. Nowadays there are four officially recognized Portuguese autochthonous horse breeds – Lusitano, Sorraia, Garrano, and Terceira Pony. The Terceira Pony is autochthonous to the Azorean archipelago and was recognized by the national authorities on January 27, 2014. These animals are believed to have descended from horses first brought from mainland Portugal to the islands during the 15th century and were selected for size and adaptation to the local conditions. These extremely hardy and well adapted horses were very important during the colonization for transportation of goods and people, for agriculture and, if necessary, for meat and milk (Luciano da Silva, 1971). Although they contributed substantially to the development of the islands, due to new agricultural practices and the introduction of horses from other origins, their importance gradually declined and therefore the sustainability of the Terceira Pony now depends on a shift toward new market needs.

The Terceira Pony has very correct and balanced proportions with physical and personality characteristics that conform to the image of a modern riding horse, making it popular for riding by children. The existing population of the Terceira Pony comprises about 100 animals living on Terceira island, many of them descending from 14 founders (six sires and eight dams) while a few horses are dispersed on the other islands, mainly Faial, Graciosa, S. Jorge, and S. Miguel. In order to preserve the existing animals and increase their number, the Association of Breeders and Friends of the Terceira Pony was established in 2010.

In general for a horse to be accepted to the studbook it has to conform to specific phenotypic characteristics. Body conformation is used in distinguishing, evaluating, and comparing breeds (Lawrence, 2001). The relationships among body dimensions also affect the horses’ beauty and performance in sports (Evans, 2000; Lawrence, 2001; Parker, 2002; Sadek et al., 2006). As the Terceira Pony has been recently recognized as a breed and the stud-book is in the process of being established, horses from other breeds can therefore still be used as founders provided that they fit the body conformation, gaits, and breeding goals set forth by the Association of Breeders and Friends of the Terceira Pony.

Nowadays, due to the reduced number of animals the Terceira Pony is evolving from deliberate crosses of specific sires and dams, which can lead to a loss of genetic diversity. Such a breeding program has to be carefully monitored to ensure that the decrease in variability does not adversely affect the beneficial characteristics of this breed. Microsatellites are accepted as the most suitable molecular markers to investigate breed genetic diversity (Takezaki and Nei, 1996) and differentiation, to estimate the genetic structure of populations (Pritchard et al., 2000) and comprise an attractive potential source of information about population histories and evolutionary processes (Aberle and Distl, 2004). The evaluation of genetic variability is one of the first steps in the process of species genetic conservation aimed at preserving both genetic variability and population viability (Bömcke et al., 2011).

The objective of the present study was to gather data to assist in management improvements and conservation planning by accessing the body conformation of the Terceira Pony by measuring 30 fully grown animals so that external breeders can match the required phenotype, estimate morphometric indices from these measures, assess biometric data and evaluate functional classification standards, and by analyzing 52 animals with 15 polymorphic microsatellite loci, that will enable quantifying the genetic variability within the breed.

## MATERIALS AND METHODS

### BIOMETRIC STUDY

For the biometric study of the Terceira Pony only fully grown animals, 11 sires and 19 dams, were measured. A total of 37 measurements (**Table 1**) were taken from the left side of the horse while standing on a horizontal surface in standard position, except for measurements of the head, with zoometric stick, and tape. Angles were measured with a protractor from the Animal Measuring System developed by ISOMED. Additionally the approximated live weight (W) was estimated from body measurement data as defined by Santos (1981).

**Table 1:** Mean and SD of body measurements and approximate live weight of dams and sires of the Terceira Pony.

Measurements		Dams	Sires
Heights (cm)	Withers^a^	127.11 ± 2.90	129.70 ± 2.36
	Sub-sternal flank^b^	72.40 ± 2.30	71.40 ± 0.89
	Back^a^	122.44 ± 3.51	123.00 ± 2.29
	Rump^a^	127.44 ± 3.52	129.40 ± 2.99
Depth (cm)	Chest^a^	54.67 ± 2.54	58.30 ± 2.66
Lengths (cm)	Head^a^	50.11 ± 3.22	49.10 ± 1.02
	Neck^a^	68.00 ± 4.39	66.50 ± 3.20
	Body^a^	127.33 ± 1.87	128.00 ± 0.71
	Barrel^a^	60.44 ± 4.45	55.50 ± 3.91
	Rearquarters^a^	38.33 ± 6.00	42.30 ± 3.56
	Shoulder^a^	50.61 ± 3.59	53.40 ± 3.13
	Humerous^a^	24.17 ± 1.41	23.70 ± 1.48
	Radius^a^	32.50 ± 1.56	33.90 ± 1.34
	Metacarpus^a^	19.61 ± 0.89	20.60 ± 1.29
	Fore phalanx^a^	10.67 ± 0.50	10.20 ± 0.27
	Small trunk^c^	89.00 ± 6.44	85.70 ± 3.60
	Pelvis^a^	27.67 ± 1.30	25.70 ± 3.38
	Femur^a^	30.94 ± 1.88	31.50 ± 1.41
	Tibia^a^	38.06 ± 1.81	40.50 ± 1.41
	Metatarsus^a^	26.33 ± 1.50	29.00 ± 1.87
	Hind phalanx^a^	10.67 ± 0.61	10.60 ± 0.42
Widths (cm)	Skull^d^	17.17 ± 1.06	16.40 ± 0.55
	Chest^a^	28.89 ± 2.22	31.00 ± 1.58
	Hips^a^	37.89 ± 1.90	38.90 ± 2.30
	Thurls^a^	35.78 ± 1.99	37.90 ± 2.36
Circumferences (cm)	Chest^a^	145.11 ± 5.23	149.20 ± 4.55
	Forelimb cannon bone^a^	15.72 ± 0083	16.10 ± 0.55
	Hindlimb cannon bone^a^	17.44 ± 0.81	17.60 ± 0.42
Angles (°)	Shoulder^a^	55.78 ± 4.41	46.80 ± 2.77
	Shoulder joint^a^	42.22 ± 4.94	41.60 ± 4.34
	Fore fetlock joint^a^	49.56 ± 3.13	53.20 ± 2.28
	Fore hoof wall^a^	48.89 ± 4.91	51.20 ± 2.28
	Croup^a^	25.44 ± 4.03	20.60 ± 1.95
	Femur^a^	52.89 ± 6.79	52.60 ± 5.73
	Hock joint^a^	56.00 ± 5.66	51.00 ± 9.17
	Hind fetlock joint^a^	52.11 ± 3.44	53.80 ± 1.48
	Hind hoof wall^a^	52.11 ± 3.44	53.40 ± 2.41

Approximate live weight (W) (kg)		244.45 ± 26.42	265.70 ± 24.22

*^a^Following the description by Zechner et al. (2001)*

*^b^Following the description by McManus et al. (2005)*

*^c^Following the description by Komosa and Purzyc (2009)*

*^d^Following the description by Solé et al. (2013).*

Based on these 37 measurements, 19 indexes (**Table 2**) were calculated to evaluate the proportions of the animals and to define its type.

**Table 2:** Conformation indices determined based on the measurements taken for dams and sires of the Terceira Pony.

Conformation indices	Dams	Sires
Body Index (BI)^a^	0.877	0.857
Dactilo-Thoracic Index (DTI)^a^	0.108	0.108
Chest Index (CI)^a^	<0	<0
Body Racio (BR)^a^	0.99	1
Quadratic Index (QI)^b^	99.83	101.32
Compact Index1 (CPI1)^a^	1.92	2.05
Compact Index 2 (CPI2)^a^	9.02	8.95
Index of Arm Trunk^c^	22.65	23.83
Index of Scapula^c^	38.82	41.17
Index of Arm (humerous)^c^	19.02	18.27
Index of Forearm (radius)^c^	25.57	26.14
Index of Metacarpus Length^c^	15.43	15.88
Index of Trunk (greater)^c^	100	98.69
Index of Trunk (smaller)^c^	70.23	66.08
Index of Femur^c^	24.28	24.34
Index of Croup^c^	30.1	33.05
Index of Chest^c^	28.82	31.3
Index of Metatarsus Lenght^c^	20.66	22.41
Index of Metacarpus Circumference^c^	12.37	12.41

*^a^Following the description by Martin-Rosset (1983)*

*^b^Following the description by Druml et al. (2008)*

*^c^Following the description by Komosa and Purzyc (2009).*

### GENETIC STUDY

A total of 52 animals, corresponding to the 14 founders (six sires and eight dams) and their descendants were genotyped in this study. Fresh blood was collected for DNA extraction, by a veterinarian and following good veterinary practices, by jugular venipuncture and placed in sterile tubes with EDTA. Tubes were kept in a refrigerator box for transport to the laboratory. DNA was extracted following a modification of the Miller et al. (1988) salting out protocol described elsewhere (Lopes, 2011).

Genotyping was performed using the 15 autosomal microsatellite markers included in the Food and Agriculture Organization/International Society of Animal Genetics Measurement of Domestic Animal Diversity panel (Hoffmann et al., 2004) [AHT4, AHT5 (Binns et al., 1995), ASB2, ASB17, ASB23 (Breen et al., 1997), HMS2, HMS3, HMS6, HMS7 (Guérin et al., 1994), HTG4, HTG6 (Ellegren et al., 1992), HTG7, HTG10 (Marklund et al., 1994), LEX33 (Coogle et al., 1996), and VHL20 (van Haeringen et al., 1994)]. Amplification was carried out in volumes of 20 μl containing 20 ng total DNA, 160 μM of each dNTP, 2–10 pmol of each primer and 1 U Taq DNA polymerase (Fermentas) in reaction buffer containing MgCl_2_. All forward primers were fluorescence labeled at the 5′ end with FAM, TET, HEX, NED, VIC, or PET, to allow multiplexing and simultaneous separation of the amplified products. Reactions were performed in a UNO II Biometra thermocycler with a first cycle of 5 min denaturation at 96°C, 40 s annealing and 40 s elongation at 72 C, followed by 35 cycles of 40 s denaturation, 40 s annealing, 40 s elongation and a final cycle of 40 s denaturation, 40 s annealing and 30 min elongation to maximize Taq DNA polymerases’ ability to catalyze non-templated nucleotide addition (Smith et al., 1995), thus minimizing the potential for genotyping error attributable to the modified “plus-A” product. The PCR products were size fractionated by capillary electrophoresis using an automated sequencer (ABI PRISM 310 Genetic Analyzer, PE Applied Biosystems) and fragment lengths were determined with the help of internal size standards (GeneScan 350 TAMRA Size Standard and GeneScan 500 LIZ Size Standard, PE Applied Biosystems).

Genetic variability was measured by estimating total number of alleles (TNA), effective number of alleles (Ne), observed (Ho), expected (He), unbiased expected heterozygosities (uHe), inbreeding coefficient (F_IS_), and average exclusion probability (PE) calculated with GenAlex 6.5 (Peakall and Smouse, 2012) and polymorphism information content (PIC) determined with Cervus 3.0.3 (Marshal et al., 1998). The software Identity (Wagner and Sefc, 1999) was used to calculate the probability of paternity exclusion (PE). Excess and deficiency of heterozygotes and deviations from Hardy–Weinberg equilibrium (Weir and Cockerham, 1984) were estimated using GENEPOP (Raymond and Rousset, 1995) using the Markov chain algorithm with 1000 de-memorization steps for every 400 batches and 1000 iterations per batch. Samples in which a single allele per locus was detected were considered homozygous genotypes, instead of heterozygous with a null allele, for the purpose of computing genetic diversity parameters.

Genetic structure of the population was inferred with the Bayesian approach of STRUCTURE (Pritchard et al., 2000). A 20,000 initial burn-in was used to minimize the effect of the starting configurations, followed by 100,000 MC iterations, as recommended by Falush et al. (2007) with 10 independent replicates each. Several sets of inferred clusters where tested to determine the most appropriate number of clusters for modeling the data. The most likely number of clusters (K) was estimated by using the maximal value of L(K) and by calculating ΔK (Evanno et al., 2005). All runs used an admixture model with correlated frequencies and the parameter of individual admixture alpha set to be the same for all clusters and with a uniform prior. To access and visualize the distribution of different animals based on genetic distances, a three-dimensional graphic using data from the first, second and third principal coordinates (PCoA) was constructed in NTSYS-PC software package (Rohlf, 1992).

## RESULTS

### BIOMETRIC STUDY

Mean values and SD of all 37 measurements, calculated with Excel, taken from the Terceira Pony dams, and sires, as well as the approximated live weight, are presented in **Table 1**. **Table 2** presents the conformation indexes determined based on the measurements taken. These indices allow comparison between breeds and studies in terms of proportions of body segments, regardless of length differences (Komosa and Purzyc, 2009).

### GENETIC STUDY

A total of 105 alleles were identified across the 15 loci, ranging from 4 for HTG7 to 11 for ASB17 and with a mean of 7 alleles per locus. The least informative locus for this population is HTG7 with the lowest values obtained for TNA, Ne, Ho, He, and PIC, and the most informative SSR marker is VHL20 with the highest TNA, Ne, He, and PIC values (**Table 3**). The total observed heterozygosity was higher than the expected heterozygosity (0.700 against 0.674, respectively), as 11 out of the 15 markers revealed observed heterozygosity values higher than the expected ones.

**Table 3:** Number of alleles (TNA), number of effective alleles (Ne), information index (I), observed heterozygosity (Ho), expected heterozygosity (He), unbiased expected heterozygosity (uHe), polymorphism information content (PIC) and heterozygote deficiency (Fis).

Locus	TNA	Ne	I	Ho	He	uHe	PIC	Fis
AHT4	8	3.337	1.423	0.725	0.700	0.707	0.654	-0.036
AHT5	7	3.463	1.449	0.824	0.711	0.718	0.667	-0.158
ASB2	8	2.869	1.384	0.596	0.651	0.658	0.615	0.085
ASB17	11	4.222	1.828	0.846	0.763	0.771	0.743	-0.109
ABS23	8	3.659	1.528	0.827	0.727	0.734	0.686	-0.138
HMS2	7	3.411	1.489	0.667	0.707	0.714	0.672	0.057
HMS3	7	3.174	1.433	0.625	0.685	0.692	0.646	0.087
HMS6	6	2.078	1.057	0.596	0.519	0.524	0.482	-0.149
HMS7	6	3.598	1.473	0.725	0.722	0.729	0.678	-0.005
HTG4	5	3.382	1.338	0.706	0.704	0.711	0.652	-0.002
HTG6	5	2.316	1.042	0.654	0.568	0.574	0.512	-0.151
HTG7	4	1.380	0.590	0.269	0.275	0.278	0.263	0.022
HTG10	7	3.367	1.483	0.809	0.703	0.711	0.666	-0.150
LEX33	8	4.125	1.654	0.769	0.758	0.765	0.727	-0.015
VHL20	8	5.520	1.830	0.857	0.819	0.827	0.794	-0.047
**Mean**	7	3.327	1.400	0.700	0.668	0.674	0.630	-0.047

Tests for Hardy–Weinberg equilibrium revealed significant deficit of heterozygotes for HMS2 (*p* = 0.0015) with the corresponding F_IS_ value of 0.057. In general all loci were highly informative with the PIC values higher than 0.5 except for HTG7. The PE was 99.99 % using this set of microsatellites, indicating its usefulness in parentage testing for this breed.

**FIGURE 1:**
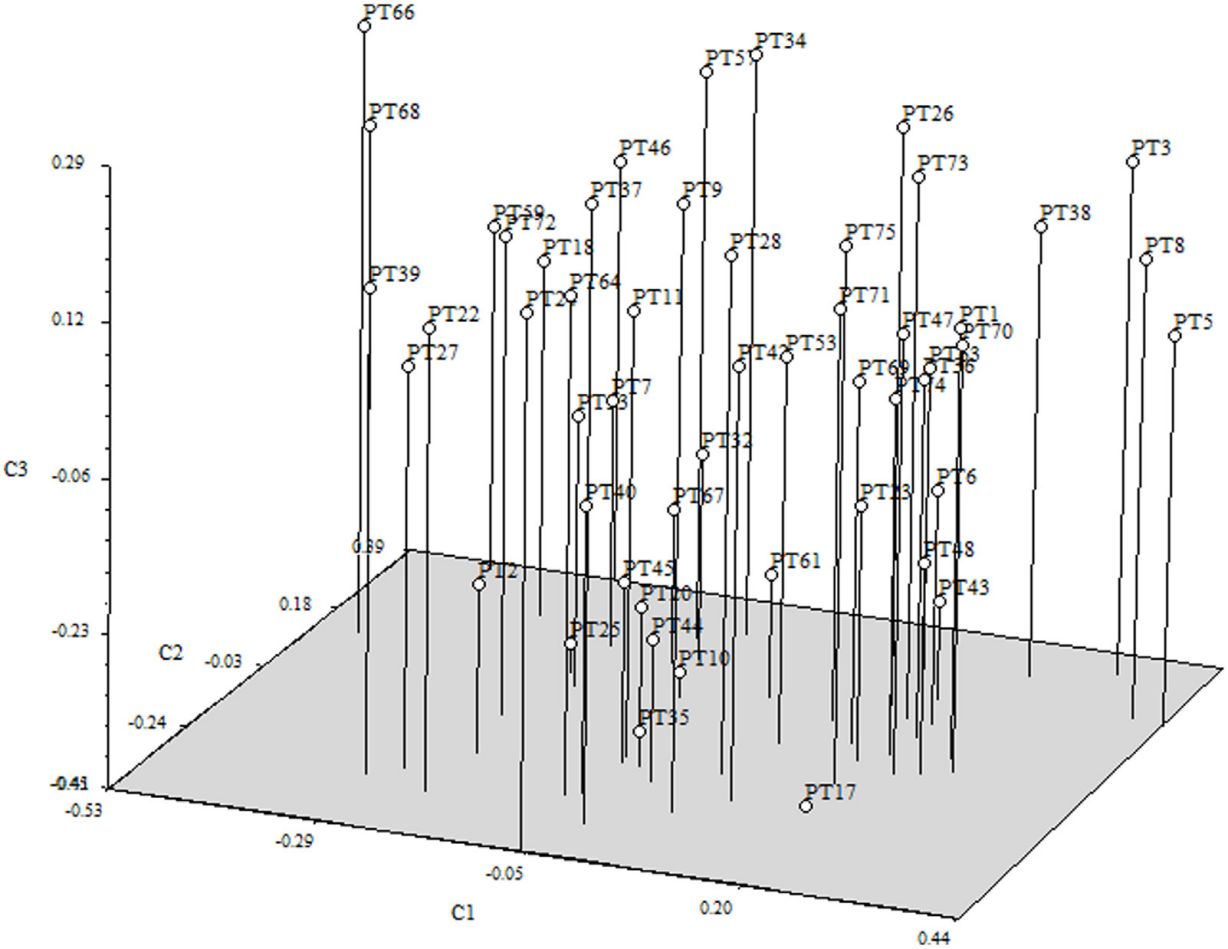
**Tri-dimensional representation of the first three axes of the principal coordinate analysis (PCoA) from the matrix of genetic distances of the Terceira Pony population**.

The Bayesian analysis carried out in STRUCTURE demonstrated that there were no genetic clusters, as average mean values of LnP(K) did not show any substantial increases when K varied from 1 to 7 (data not shown). This result was supported by the PCoA based on the matrix of individual genotypes where no sub-grouping is observed, though a few individuals, all descending from the same founder mare (PT8), appear at the right margins of the figure. In the PCoA the first three PCoAs explain 28.20% (axis 1 = 11.25%; axis 2 = 8.78%; axis 3 = 8.17%) of the variation in the data (**Figure 1**)

## DISCUSSION

The objective of this work was to determine the morphotype of the Terceira Pony and to analyze its genetic structure.

Horse breeding aims at influencing functional conformation for the improvement of traits such as sport performance. Identifying patterns of equine anatomy for different horse breeds could be helpful in predicting how successful the animals will be in performing different tasks. Part of the beauty of the Terceira Pony depends on its body conformation, body measurements and the relationships among the dimensions; therefore, metric features of the exterior can become a selection tool for the Terceira Pony (Koenen et al., 2004).

Among the standard measurements used in horse breeding, the most significant exterior variable is height at withers (Komosa and Purzyc, 2009). An average of height at withers of 128.00 cm for the population under study classifies these horses as ponies (Hendricks, 2007).

The present study indicated that from a morphological and zoometric point of view the Terceira Pony may be regarded as a mediline (BI = 0.877 for dams and 0.857 for sires; Oom and da Costa Ferreira, 1987), eumetric (DTI = 0.108; Cabral et al., 2004), elipometric (*W* < 350 kg; McManus et al., 2008), dolichocephalic and “far from ground” (CI < 0; Ribeiro, 1988; McManus et al., 2008) animal. Also the BR and QI indicate that these animals are well proportioned, with withers height, croup height, and body length approximately equal. Due to these proportions the Terceira Pony can therefore be considered as a well-proportioned saddle horse suitable for sports (Zamborlini, 2001).

The CPI defines how compact horses are and gives an indication on their aptitude for traction (McManus et al., 2005, 2008). Values obtained for the Terceira Pony are contrasting: while CPI1 showed that animals were more adapted for riding, CPI2 indicated animals adapted for light traction. The difference observed between the two indices reveals the versatility and potential of these animals, which is also supported by the variation of the angle of the shoulder. Shoulders should be efficient to transform speed into driving force transmitted by the hind limbs and therefore variations of the shoulder angle may be an indication of the potential use of the animals (Camargo and Chieffi, 1971). The longer and more obliquely positioned scapula results in a longer and more swinging gait. For the Terceira Pony the shoulder angle of sires ranges from 45 to 55° and of dams is 55° suggesting that sires can be used for draft and saddle and dams for racing (Camargo and Chieffi, 1971).

The long bones that form the limbs and thus affect height are of special importance, not only to the appearance of an animal but also to the quality of gaits and practical predispositions. From the anatomical point of view, hind limbs are used to start a stride and fore limbs are used mainly to support the body mass during movement (Mawdsley et al., 1996). The Terceira Pony has a long hind limb with indices of the metacarpus length and the metatarsus length higher than those reported for other Pony breeds (Komosa and Purzyc, 2009); this predisposition was previously associated with jumping in Polish Half-bred horses (Komosa et al., 2013).

Longer croups are desirable in racing, jumping and also in marchers and are associated with elongated and stronger muscles capable of powerful contractions necessary for speed and which facilitate propulsion (Jones, 1987). A short croup is tolerated only in draft horses, but this reduction in length must be compensated by greater muscle development (Nascimento, 1999). Also the slope of the croup influences the fitness of the horse. A croup with a horizontal direction (12–25°) is conducive to speed, inclined (25–35°) is suitable for light traction, jumping and riding, oblique (35–45°) should only be tolerated for heavy traction, and too steep (45 and 55°) is always undesirable (Nascimento, 1999). For the Terceira Pony, while males have croups with a horizontal direction (20.60°) favorable for speed, females stand out from the males for presenting an inclined croup (25.44°) suitable for jumping and riding.

According to Komosa et al. (2013) among the indices used for describing the differences in the exterior conformation of horses, the scapula index deserves particular attention. The scapula belongs to the flat bones and plays a considerable role in the movement of a horse (Komosa and Purzyc, 2009). By comparison with other Pony horses, the Hucul and the Konik (Komosa and Purzyc, 2009), the Terceira Pony showed generally lower values for the following indices: arm trunk, arm, forearm, greater trunk, smaller trunk, femur, and metacarpus circumference. The only exceptions were for the indices of scapula, chest, and croup where the Hucul horses showed slightly lower values; and for the index of metacarpus and metatarsus lengths where the Terceira Pony showed higher values than the other two breeds. The scapula, greater trunk, smaller trunk, and metacarpus circumference indices of the Terceira Pony were closer to those of the Polish Half-bred horse and the Thoroughbred (Komosa et al., 2013) than the Hucul and Konik breeds. Higher values of greater trunk index were reported for the Pantaneiro horse (McManus et al., 2008) and lower for the Alter Real (Oom and da Costa Ferreira, 1987) and the Mangalarga Marchador (Cabral et al., 2004) when compared with the Terceira Pony.

Based on this study, it can be stated that due to its height at withers, the Terceira Pony is different from the Thoroughbred and Polish Half-bred horses, but its exterior conformation is more similar to these riding breeds than to other primitive ponies like the Hucul and Konik horses.

Although these results may seem unexpected, the genetic background of the Terceira Pony is closer to the Polish Half-bred horse and to the Thoroughbred than to the Konik and Hucul horses. The Terceira Pony is believed to be a representative of the horses living in the Iberian Peninsula during the Portuguese and Spanish discoveries, that contributed to the development of many other European modern horse breeds that were later introduced and dispersed throughout the Americas, founding numerous breeds in the new world (Rodero et al., 1992; Luís et al., 2007; Lopes, 2011). A preliminary study conducted with 64 worldwide horse breeds including the Terceira Pony showed closest genetic similarity with breeds form Iberian origin (Lopes et al., 2011).

The genetic analysis showed that the Terceira Pony presents levels of genetic diversity similar to other, older breeds from the Iberian Peninsula, breeds from South America of Iberian origin, as well as breeds from Asia, Europe and North and South America (Luís et al., 2007; Felicetti et al., 2010; Cothran et al., 2011; Lopes, 2011). However, allelic frequencies are heterogeneous and equal to or higher than 40% for all loci except VHL20. This may be explained by the different founder lineages that the actual population may have. A census made in 2001 in Terceira island identified 144 horses with a height at withers equal or lower than 139 cm out of 726 horses (Braga, personal communication) which are believed to be the founders of the actual population. Ponies with the same phenotypic characteristics were also identified in other islands and recently introduced in the herd, who at the time this work was conducted, had few or no descendants. Nevertheless, although some founders were more represented than others, no sub division of the population was observed by either analysis methods, a result typical of a homogeneous population. According to Pedersen (1999) and Proschowsky et al. (2003) lower values for genetic diversity were expected as until now phenotypic selection has been based on a few isolated sires and dams.

The Terceira Pony has been associated with husbandry and social activities of the people from Terceira island over 100s of years. Presumably the animals that were more robust and tractable and of the “desirable type” have been bred by farmers. Therefore the Terceira Pony is a breed autochthonous of the Azorean archipelago and very well adapted to the local conditions. However, with a reduced number of animals, it still sustains relatively high genetic diversity that needs to be taken into account in future breeding strategies, for conservation purposes and in the management of the studbook to avoid genetic erosion.

**FIGURE 2:**
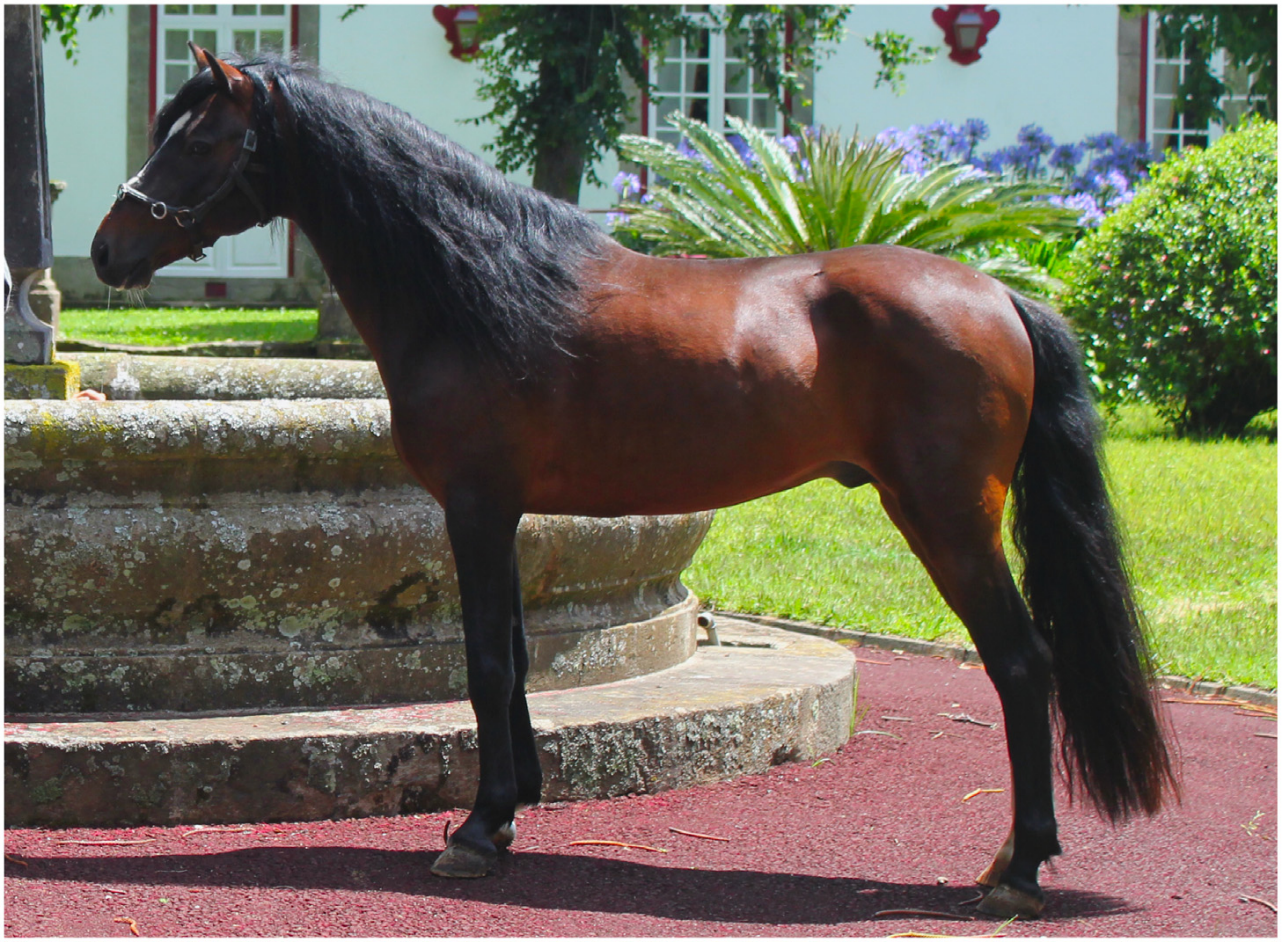
**Trovão – Founder stallion of the Terceira Pony (height at withers 1.29 m)**.

The data presented in this study define this horse breed as well-proportioned with a small and narrow head, a long neck well placed between the long shoulders and leaving the withers without any convexity. The overall average of biometric variables and trends classify the Terceira Pony as having small shape, low weight, shortline, eumetric, or well balanced with strong and resistant legs considered far from ground (**Figure 2**). It is a fast animal, smart, extremely docile and easy to manage and therefore ideal for teaching equestrian sports to young children and for physical therapy or work with disabled people. Due to its phenotypic homogeneity in body conformation, balanced gaits, personality, and cultural importance and geographical location (FAO, 1999) the Terceira Pony was recently recognized as a breed. However, as the Terceira Pony still has an open stud book allowing the entrance of horses from other origins with a specific morphological type, and as breeds tend to change based on the function they are breed for, these results will serve as baseline data for future follow-up studies of the breed. The standard established for the Terceira Pony and the genetic data presented in this work are therefore of utmost importance, as the harmonization of selection and preservation is difficult and they may be opposed to one other (Bodó, 1990).

## AUTHOR CONTRIBUTIONS

ACM conceived the experiment. HR and VC collected measurements and blood. MSL performed the genetic analysis. MSL, DM, and SXB analyzed the data. MSL, SXB, and ACM wrote the manuscript. All authors read the manuscript and agreed to submit it.

## Conflict of Interest Statement

The authors declare that the research was conducted in the absence of any commercial or financial relationships that could be construed as a potential conflict of interest.

## References

[B1] Aberle K. S. Distl O. (2004). Domestication of the horse: results based on microsatellite and mitochondrial DNA markers. *Arch. Tierz.* 47 517–535.

[B2] Binns M. M. Holmes N. G. Holliman A. Scott A M. (1995). The identification of polymorphic microsatellite loci in the horse and their use in thoroughbred parentage testing. *Br. Vet. J.* 151 9–15 10.1016/S0007-1935(05)80057-0 7735875

[B3] Bodó I. (1990). “Methods and experiences with in situ preservation of farm animals,” in *Animal Genetic Resources*, ed. Wiener G. (Rome: FAO Animal Production and Health Paper 80) 85–102.

[B4] Bömcke E. Gengler N. Cothran E. G. (2011). Genetic variability in the Skyros pony and its relationship with other Greek and foreign horse breeds. *Genet. Mol. Biol.* 34 68–76 10.1590/S1415-47572010005000113 21637546 PMC3085377

[B5] Breen M. Lindgren G. Binns M. M. Norman J. Irvin Z. Bell K. (1997). Genetical and physical assignments of equine microsatellites-first integration of anchored markers in horse genome mapping. *Mamm. Genome* 8 267–273 10.1007/s003359900407 9096108

[B6] Cabral G. C. de Almeida F. Q. Quirino C. R. de Azevedo P. C. N. Batista Pinto L. F. Santos E. M. (2004). Morphometric evaluation of Mangalarga Marchador horse: conformation index and body proportions. *R. Bras. Zootec.* 33 1798–1805 10.1590/S1516-35982004000700018

[B7] Camargo M. X. Chieffi A. (1971). *Ezoognózia*. São Paulo: Instituto de Zootecnia 320.

[B8] Coogle L. Reid R. Bailey E. (1996). Equine dinucleotide repeat loci from LEX025 to LEX033. *Anim. Genet.* 27 289–290 10.1111/j.1365-2052.1996.tb00500.x 8856936

[B9] Cothran E. G. Canelon J. L. Luís C. Conant E. Juras R. (2011). Genetic analysis of the Venezuelan Criollo horse. *Genet. Mol. Res.* 10 2394–2403 10.4238/2011.October.7.1 22002132

[B10] Druml T. Baumung R. Sölkner J. (2008). Morphological analysis and effect of selection for conformation in the Noreiker draught horse population. *Livest. Sci.* 115 118–128 10.1016/j.livsci.2007.06.015

[B11] Ellegren H. Johansson M. Sandberg K. Andersson L. (1992). Cloning of highly polymorphic microsatellites in the horse. *Anim. Genet.* 23 133–142 10.1111/j.1365-2052.1992.tb00032.x 1443772

[B12] Evanno G. Regnaut S. Goudet J.(2005). Detecting the number of clusters of individuals using the software STRUCTURE: a simulation study. *Mol. Ecol.* 14 2611–2620 10.1111/j.1365-294X.2005.02553.x 15969739

[B13] Evans J. W. (2000). *Horses: A Guide to Selection, Care and Enjoyment*. New York: W. H. Freeman and Company 896.

[B14] Falush D. Stephens M. Pritchard J. K. (2007). Inference of population structure using multilocus genotype data: dominant markers and null alleles. *Mol. Ecol. Notes* 7 574–578 10.1111/j.1471-8286.2007.01758.x 18784791 PMC1974779

[B15] FAO. (1999). *The Global Strategy for the Management of Farm Animal Genetic Resources.* Rome: Executive Brief 43.

[B16] Felicetti M. Lopes M. S. Verini-Supplizi A. da Câmara Machado A. Silvestrelli M. Mendonça D. (2010). Genetic diversity in the Maremmano horse and relationships with other European breeds. *Anim. Genet.* 41 53–55 10.1111/j.1365-2052.2010.02102.x 21070276

[B17] Guérin G. Bertaud M. Amiques Y. (1994). Characterization of seven new horse microsatellites: HMS1, HMS2, HMS3, HMS5, HMS6, HMS7 and HMS8. *Anim. Genet.* 25 62 10.1111/j.1365-2052.1994.tb00072.x 8161034

[B18] Hendricks B. L. (2007). *International Encyclopedia of Horse Breeds.* Norman: University of Oklahoma Press 496.

[B19] Hoffmann I. Ajmone Marsan P. Barker J. S. F. Cothran E. G. Hanotte O. Lenstra J. A. (2004). “New MoDAD marker sets to be used in diversity studies for the major farm animal species: recommendations of a joint ISAG/ FAO working group,” in *Proceedings of the 29th International Conference on Animal Genetics* 123 Tokyo.

[B20] Jones W. E. (1987). *Genética e Criação de Cavalos*. São Paulo: Roca 666.

[B21] Koenen E. P. C. Aldridge L. I. Philipsson J. (2004). An overview of breeding objectives for warmblood sport horses. *Livest. Prod. Sci.* 88 77–84 10.1016/j.livprodsci.2003.10.011

[B22] Komosa M. Frackowiak H. Purzyc H. Wojnowska M. Gramacki A. Gramacki J. (2013). Differences in exterior conformation between primitive, Half-bred, and Thoroughbred horses: anatomic-breeding approach. *J. Anim. Sci.* 91 1660–1668 10.2527/jas.2012-5367 23345554

[B23] Komosa M. Purzyc H. (2009). Konik and Hucul horses: a comparative study of exterior measurements. *J. Anim. Sci.* 87 2245–2254 10.2527/jas.2008-1501 19329479

[B24] Lawrence L. A. (2001). *Horse Conformation Analysis.* Pullman, WA: Washington State University 10.

[B25] Lopes M. S. (2011). *Molecular Tools for the Characterisation of the Lusitano Horse.* Ph.D. thesis, University of Azores Azores 216.

[B26] Lopes M. S. Cothran G. Juras R. Mendonça D. da Câmara Machado A. (2011). “Origin of the endangered Terceira pony assessed by microsatellites and mitochondrial DNA sequence variation,” in *Proceedings of the Ninth Dorothy Russell Havemeyer International Equine Genome Mapping Workshop* Chaska, MN.

[B27] Luciano da Silva M. (1971). *Portuguese Pilgrims and Dighton Rock* Chap. 11. Available at: http://www.dightonrock.com/pilgrim_chapter_11.htm

[B28] Luís C. Juras R. Oom M. M. Cothran E. G. (2007). Genetic diversity and relationships of Portuguese and other horse breeds based on protein and microsatellite loci variation. *Anim. Genet.* 38 2–27 10.1111/j.1365-2052.2006.01545.x 17257184

[B29] Marklund S. Ellegren H. Eriksson S. Sandberg K. Andersson L. (1994). Parentage testing and linkage analysis in the horse using a set of highly polymorphic microsatellites. *Anim. Genet.* 25 19–23 10.1111/j.1365-2052.1994.tb00050.x 8161016

[B30] Marshal T. C. Slate J. Kruuk L. Pemberton J. M. (1998). Statistical confidence for likelihood-based paternity inference in natural populations. *Mol. Ecol.* 7 639–655 10.1046/j.1365-294x.1998.00374.x 9633105

[B31] Martin-Rosset W. (1983). Particularités de la croissance et du développment du cheval. *Ann. Zootec.* 32 373–380 10.1051/animres:19830108

[B32] Mawdsley A. Kelly E. P. Smith F. H. Brophy P. O. (1996). Linear assessment of the thoroughbred horse: an approach to conformation evaluation. *Equine Vet. J.* 28 461–467 10.1111/j.2042-3306.1996.tb01618.x 9049495

[B33] McManus C. M. Falcão R. A. Spritze A. Costa D. Louvandini H. Dias L. T. (2005). Caracterização morfológica de equinos de raça Campeiro. *R. Bras. Zootec.* 34 1553–1562 10.1590/S1516-35982005000500015

[B34] McManus C. M. Santos S. A. da Silva J. A. Louvandini H. Avreu U. G. P. Sereno J. R. B. (2008). Body indices for the Pantaneiro horse. *Braz. J. Vet. Res. Anim. Sci.* 45 362–370.

[B35] Miller S. A Dykes D. D. Polesky H. F. (1988). A simple salting out procedure for extracting DNA from human nucleated cells. *Nucleic Acids Res.* 16 1215 10.1093/nar/16.3.1215 PMC334765 3344216

[B36] Nascimento J. F. (1999). *Mangalarga Marchador: Tratado Morfofuncional*. Belo Horizonte: Associação Brasileira dos Criadores de Cavalos Mangalarga Marchador 577.

[B37] Oom M. M. da Costa Ferreira J. (1987). Estudo biométrico do cavalo Alter. *Rev. Port. de Ciênc. Vet.* 82 101–148.

[B38] Parker R. (2002). *Equine Science.* New York: Thomson Delmar Learning 688.

[B39] Peakall R. Smouse P. E. (2012). GenAlEx 6.5: genetic analysis in Excel. Population genetic software for teaching and research-an update. *Bioinformatics* 28 2537–2539 10.1093/bioinformatics/bts460 22820204 PMC3463245

[B40] Pedersen N. C. (1999). A review of immunologic diseases of the dog. *Vet. Immunol. Immunopathol.* 69 251–342 10.1016/S0165-2427(99)00059-8 10507310 PMC7119806

[B41] Pritchard J. K. Stephens M. Donnelly P. (2000). Inference of population structure using multilocus genotype data. *Genetics* 155 945–959. 10835412 10.1093/genetics/155.2.945 PMC1461096

[B42] Proschowsky H. F. Rugbjerg H. Ersboll A. K. (2003). Mortality of purebred and mixed-breed dogs in Denmark. *Prev. Vet. Med.* 58 63–74 10.1016/S0167-5877(03)00010-2 12628771

[B43] Raymond M. Rousset F. (1995). GENEPOP (Version 1.2): population genetics software for exact tests and ecumenicism. *J. Hered.* 86 248–249.

[B44] Ribeiro D. B. (1988). *O Cavalo: Raças, Qualidade e Defeitos*. São Paulo: Editora Globo 318.

[B45] Rodero A. Delgado J. V. Rodero E. (1992). Primitive Andalusian livestock and their implications in the discovery of America. *Arch. Zootec.* 41 383–400.

[B46] Rohlf F. J. (1992). *Numerical Taxonomy and Multivariable Analysis System (Version 2.11f).* New York: Applied Biostatistics Inc. 44.

[B47] Sadek M. H. Al-Aboud A. Z. Ashmawy A. A. (2006). Factor analysis of body measurements in Arabian horses. *J. Anim. Breed. Genet.* 123 369–377 10.1111/j.1439-0388.2006.00618.x 17177691

[B48] Santos R. F. (1981). *O Cavalo de Sela Brasileiro e Outros Equídeos*. Botucatu: Editora Varela 288.

[B49] Smith J. R. Carpten J. D. Brownstein M. J. Ghosh S. Magnuson V. L. Gilbert D. A. (1995). Approach to genotyping errors caused by nontemplated addition by Taq DNA Polymerase. *Genome Res.* 5 312–317 10.1101/gr.5.3.312 8593617

[B50] Solé M. Gómez M. D. Molina A. Peña F. Valera M. (2013). Analyses of conformational performance differentiation among functional breeding goals in the Menorca horse breed. *Arch*. *Tierz.* 37 367–379 10.7482/0003-9438-56-037

[B51] Takezaki N. Nei M. (1996). Genetic distance and reconstruction of phylogenetic trees from microsatellite DNA. *Genetics* 144 389–399. 8878702 10.1093/genetics/144.1.389 PMC1207511

[B52] van Haeringen H. Bowling A. T. Scott M. L. Lenstra J. A. Zwaagstra K. A. (1994). A highly polymorphic horse microsatellite locus: VHL20. *Anim. Genet.* 25 207 10.1111/j.1365-2052.1994.tb00129.x 7943974

[B53] Wagner H. W. Sefc K. M. (1999). *IDENTITY 4.0. Centre for Applied Genetics.* Vienna: University of Agricultural Sciences.

[B54] Weir B. S. Cockerham C. C. (1984). Estimating F-statistics for the analysis of population structure. *Evolution (N. Y.)* 38 1358–1370. 10.1111/j.1558-5646.1984.tb05657.x 28563791

[B55] Zamborlini L. C. (2001). *Estudo Genético-Quantitativo da Raça Mangalarga Marchador*. Ph.D. thesis, *Escola de Veterinária, Universidade Federal de Minas Gerais, Belo Horizonte* 39.

[B56] Zechner P. Zohman F. Sölkner J. Bodo I. Habe F. Marti E. (2001). Morphological description of the Lipizzan horse population. *Livest. Prod. Sci.* 69 163–177 10.1016/S0301-6226(00)00254-2

